# Clinical Features of Hypoxemia Due to Infection Under Home-Based Medication During the COVID-19 Pandemic Period

**DOI:** 10.7759/cureus.34178

**Published:** 2023-01-25

**Authors:** Kentaro Nagaoka, Tomoya Iida, Nagomi Ito, Naoka Okamura, Michio Iida, Yoshiki Wada, Masahiro Hirano, Shu Nishikawa, Hitoshi Kawasuji, Yoshihiro Yamamoto, Hideo Yoshizaki

**Affiliations:** 1 Clinical Infectious Diseases, Toyama University Graduate School of Medicine and Pharmaceutical Sciences, Toyama, JPN; 2 Home-Care Medicine, Sapporo Home-Care Clinic Soyokaze, Sapporo, JPN; 3 Home-Care Medicine, Sapporo Zaitaku-iryo Clinic, Sapporo, JPN

**Keywords:** supportive and palliative care, hypoxemia, covid-19, pneumonia, home-based medication

## Abstract

Background: Despite the growing demand for home-based medication during the COVID-19 pandemic period, there remains scarce evidence of hypoxemic infection in home-care settings. In this study, we investigated the clinical features of hypoxemic respiratory failure due to infection during the period under home-based medication (defined as ‘home-care-acquired infection’).

Methods: This retrospective observational study enrolled patients with home-care-acquired infection, other than COVID-19, in two home-care clinics in Sapporo, Japan, between April 2020 and May 2021 (the early phase of the COVID-19 pandemic). The participants were divided into two groups according to whether they required additional home oxygen therapy, and were compared to assess the predictors of hypoxemic respiratory failure. Furthermore, the clinical features were compared with those in patients aged >60 years with COVID-19 who were admitted to Toyama University Hospital during the same period.

Results: A total of 107 patients with home-care-acquired infections were included in the study (median age, 82 years). Twenty-two patients required home oxygen therapy, and 85 did not. Thirty-day mortality rates were 32% and 8%. Among the patients in the hypoxemia group, none had desired a care-setting transition, following the advanced care planning. Multivariable logistic regression analysis showed that initial antibiotic treatment failure and malignant disease were independently associated with hypoxemic respiratory failure (odds ratio, 7.28 and 7.10; p=0.023 and p<0.005, respectively). In comparison with hypoxemia in the COVID-19 cohort, the lower incidence of febrile co-habitants and earlier onset of hypoxemia were significant in those due to home-care-acquired infection.

Conclusion: This study demonstrated that hypoxemia due to home-care-acquired infection was characterized by distinct features, possibly different from those due to COVID-19 in the early pandemic period.

## Introduction

Home-based medication, primary care, and palliative care are alternative approaches for in-hospital medication, which have been applicable for the elderly or patients with end-stage chronic diseases, including those requiring home oxygen therapy [1,2]. In comparison with in-hospital medication, home-based medication has several advantages, such as suppressing deterioration of motor and cognitive functions by avoiding hospitalization-associated disability and promoting quality of life in palliative medication [3-5]. Despite the growing demand for home-based medication, there remains scarce evidence of infection or respiratory failure in these settings [6-8].

Since the first outbreak of coronavirus disease (COVID-19), this pandemic has affected the capacity of local and regional healthcare systems worldwide, resulting in temporal exhaustion of in-hospital medical services [9,10]. In Japan, the recurrent outbreak of COVID-19 began in February 2020 and continued until January 2023. During the period when vaccination against COVID-19 was unavailable (between February 2020 and May 2021, which corresponded to the early pandemic era of COVID-19), hospitalization was highly restricted due to insufficient medical resources, which also largely affected the management of home-based medication.

Fortunately, none developed COVID-19 during this period among those who had received home-based medication at our clinics. However, acute onset fever imposed a heavy burden on clinical management, since immediate ruling out of COVID-19 was difficult. Due to the nature of the home-based medication, most laboratory examinations were conducted in external institutions, and at least 48 hours were required to confirm the result of polymerase chain reaction (PCR) detecting severe acute respiratory syndrome coronavirus 2 (SARS-CoV-2). Moreover, the insufficiency of evident information on infection in the home-care settings complicated clinical decisions when encountering acute febrile patients, particularly those with hypoxemia.

Based on these experiences, we noted the urgent necessity to examine the clinical features of home-care-acquired infection other than COVID-19, particularly in patients who developed hypoxemia, which would be an important diagnostic aid for the clinical practice in home-care settings during the era of emerging infection-pandemic, including COVID-19. Therefore, this study aimed to investigate the predictors of hypoxemia in patients who had received home-based medicine (defined as ‘home-care-acquired infection’) and compare them with those associated with COVID-19 during the early pandemic period. The primary endpoint of this study was to validate the predictive factors of hypoxemia due to home-care-acquired infection. The secondary endpoint was to extract the clinical features of these infections in comparison with those of COVID-19.

## Materials and methods

Study design

This retrospective study included all consecutive patients receiving home-based medication provided by two clinics in Sapporo, Japan, from April 2020 to May 2021. For comparison, we also included patients who were diagnosed with COVID-19 confirmed by quantitative reverse transcription PCR (RT-qPCR) and admitted to Toyama University Hospital, Toyama, Japan, during the same period. This observational, non-interventional analysis of medical records was approved by the Human Subjects Review Committee of Toyama University Hospital (R2021077). Informed consent was waived owing to the nature of the study design.

Study participants and protocol

As per the Japanese medical system, home-based medication was defined as follows: (1) medication provided to either individual residents or healthcare facilities, (2) provided by physicians from the medical institution located within 15 km from the dwellings. No oxygen administration system was provided in either residence of the participants. In the home-based medical care system, patients receive regular doctor visits once or twice per month and can require additional visits in case of medical emergencies [11]. Once the patient develops an emergent event, immediate medications at home can be supplied within a few hours, including intravenous antibiotic administration and home oxygen therapy ranging between 0 and 14 L/min of oxygen supply.

In this study, participants who developed acute onset fever and received antibiotics were included for further analysis. Acute onset fever was defined as an elevation of body temperature over 1.5 degrees Celsius within a day, and fever decline was determined when body temperature was reduced to the usual state. In our clinics, almost all the patients received certain antibiotics immediately after the onset of fever during the study period, in order to avoid admission to the hospital and to differentiate from COVID-19. Because the immediate identification of SARS-CoV-2 by PCR was unavailable during the study period in our region, we had to rule out COVID-19 mainly by the clinical feature, such as the rapid recovery response after antibiotic administration, with aid of rapid antigen test against SARS-CoV-2 when febrile state prolonged. The febrile patients who did not receive any antibiotics were excluded since fever in those cases declined before the doctor's visit.

The participants were divided into two groups according to the presence of hypoxemic respiratory failure (SpO_2_<93% at rest), which required additional oxygen therapy after the onset of infection. The following data were collected from the medical records: age, sex, underlying disease, presence of febrile co-habitant, clinical diagnosis, duration of fever, antibiotic treatment, and prognosis until 30 days after onset. Clinical diagnosis was determined by each physician according to previously defined criteria based on a physical examination [7]. Since the radiological examination was unavailable under the home-care settings, pneumonia or bronchitis were integrated as lower respiratory tract infections. Initial treatment failure was defined in cases that required other antibiotics when infection deteriorated after initial antibiotic administration.

Comparison of clinical features in patients with home-care-acquired infections with those in COVID-19 patients

To compare clinical features in patients with a hypoxemic infection under home-based medication with those in patients with COVID-19, the medical records of the COVID-19 cohort were also investigated. As recommended elsewhere [12], the initiation of oxygen therapy for respiratory failure due to COVID-19 in our institution was also determined when respiratory failure was extended to SpO_2_ ≤93% at rest under room air. In this study, participants with COVID-19 over 60 years of age were selected for the analysis in order to reduce the confounding effect of age among the two cohorts.

Statistical analysis

Background factors are expressed as median (interquartile range) or number (percentage). To evaluate differences between the two groups, the Wilcoxon test and Pearson’s chi-squared test were used to compare continuous and nominal variables, respectively. Statistical significance was set at p<0.05. Predictors associated with hypoxemic respiratory failure due to home-care-acquired infection or COVID-19 were determined using a logistic regression model. Candidate predictors to enter the logistic multivariate regression were variables with p<0.05, as determined in univariate analysis. JMP Pro 16 (SAS Institute, Cary, NC) was used for statistical analysis.

## Results

Clinical features, treatment, and prognosis of the patients included in this study

**Figure 1:**
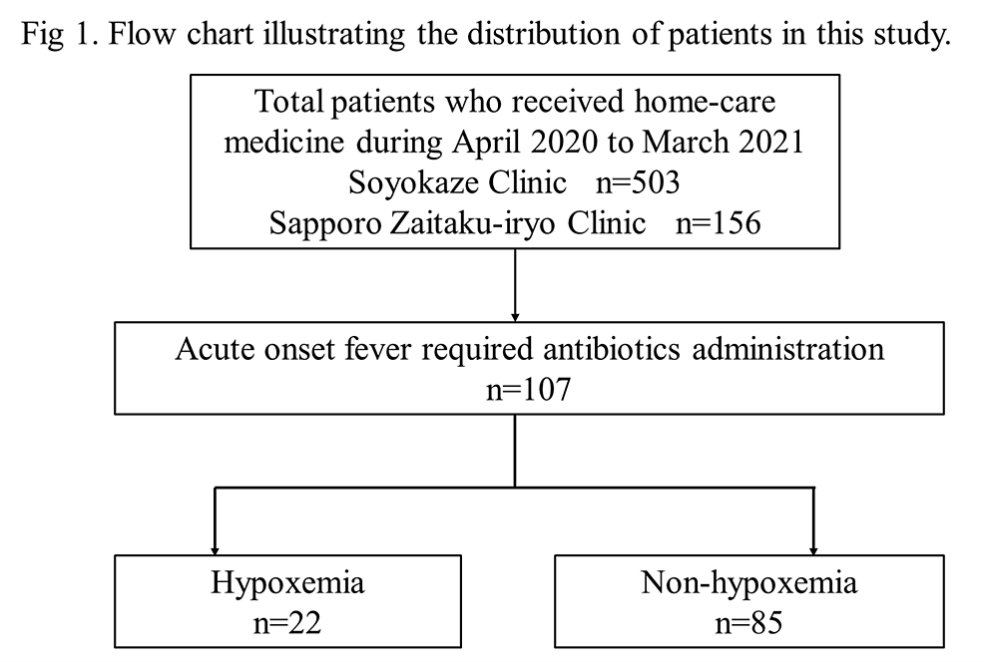
Flow chart illustrating the distribution of patients in this study.

A flow chart illustrating the patient inclusion in two different groups is shown in Figure 1.

The incidence of infection requiring antibiotic administration was 16.2% per year.

Table 1 summarizes the clinical characteristics of patients with home-care-acquired infections. For most of the subjects, the clinical features were compatible with bacterial infection, which presented certain recovery responses after the antibiotic administration. Ninety-nine patients (93% of the whole cohort) improved from the infection after the antibiotic administration. Sixteen patients received the examination for COVID-19 because of the prolonged fever; the examination was performed using 10 with rapid antigen test, and six with PCR, which revealed negative results for all the cases. At least, symptomatic COVID-19 was ruled out for all the patients with home-case acquired infections in this study.

**Table 1:** Clinical characteristics of the patients with home-care-acquired infection. Continuous variables are presented as median (interquartile range (IQR) 25-75). Categorical variables are presented as numbers (percentages).

	Total (n=107)	Hypoxemia (n=22)	Non-hypoxemia (n=85)	p
Age, years	82 (73-89)	76 (71-81)	81 (73-89)	0.008
Sex (male/female)	52/55 (48.6% vs 41.4%)	13/9 (59% vs 41%)	39/46 (45.9% vs 54.1%)	0.269
Individual home/Healthcare home	43/64 (40.2% vs 59.8%)	12/10 (54.5% vs 45.6%)	31/54 (36.5% vs 63.5%)	0.123
Underlying disease				
Dementia	50 (47%)	6 (27%)	44 (52%)	0.040
Malignant tumor	29 (27%)	12 (55%)	17 (20%)	0.001
Degenerative neurological disorder	22 (21%)	6 (27%)	16 (19%)	0.382
Diabetes mellitus	13 (12%)	3 (14%)	10 (12%)	0.811
Hypertension	34 (32%)	5 (23%)	29 (34%)	0.306
Febrile co-habitant in simultaneous period	3 (3%)	0 (0%)	3 (4%)	0.371
Clinical diagnosis				
Lower respiratory tract infection	55 (44%)	18 (82%)	37 (44%)	0.001
Urinary tract infection	35 (33%)	2 (9%)	33 (39%)	0.008
Biliary tract infection	5 (5%)	1 (5%)	4 (5%)	0.975
Focus unknown/others	5 (5%)	1 (5%)	4 (5%)	0.975
Duration of fever (days)	2 (2-4)	3 (2-5)	2 (2-4)	0.245
Fever declined within 72 hours	77 (73%)	11 (50%)	66 (78%)	0.010

Age and the presence of dementia/malignant tumor, clinically diagnosed as lower respiratory tract infection or urinary tract infection, were significantly different between the two groups. Febrile co-habitant was found only in three cases without hypoxemia; all the patients dwelled in the healthcare facility. Fever decline within 72 hours after onset was achieved in 78% of the patients in the non-hypoxemia group, which was significantly higher than that in the hypoxemia group (50%).

The treatment and outcomes in patients with home-care-acquired infection are summarized in Table 2. A total of 107 patients developed infections under home-based medication; 98% were treated without admission to the hospital, 91% received emergency doctor visits within 24 hours after fever onset, and 91% were improved with antibiotic administration. For initial antibiotic administration, intravenous administration of ceftriaxone was frequently used in home-care settings, followed by oral administration of quinolone. In the healthcare facility with a home-visit nurse station, tazobactam-piperacillin was administered twice or thrice daily. In order to avoid admission, 41% of hypoxemia was treated using steroids for a short period until oxygenation was improved, which amounted to three to five days. Death due to deterioration of infection was observed in 18% of the patients in the hypoxemia group and 5% in the non-hypoxemia group, while 14% of the patients in hypoxemia and 3% in non-hypoxemia groups died due to the progression of underlying disease after improvement of infection.

**Table 2:** Treatment and outcomes in the patients with home-care-acquired infection. Categorical variables are presented as numbers (percentages). CTRX: ceftriaxone; GRNX: garenoxacin; i.v.: intravenous; LVFX: levofloxacin; STFX: sitafloxacin; TAZ/PIPC: tazobactam piperacillin

	Total (n=107)	Hypoxemia (n=22)	Non-hypoxemia (n=85)	p
Antibiotic treatment				
CTRX iv +LVFX/STFX/GRNX	25 (23%)	8 (36%)	17 (20%)	0.106
LVFX po/iv	41 (38%)	1 (5%)	40 (47%)	<0.001
STFX po	11 (10%)	4 (18%)	7 (8%)	0.171
GRNX po	17 (16%)	6 (27%)	11 (13%)	0.101
TAZ/PIPC div (q12h or q8h)	8 (7%)	3 (14%)	5 (6%)	0.218
Others	5 (5%)	0 (0%)	5 (6%)	0.371
Initial antibiotics i.v.	33 (31%)	11 (50%)	22 (26%)	0.029
Initial treatment failure	12 (11%)	6 (27%)	6 (7%)	0.007
Treatment other than antibiotics				
Steroid therapy	16 (15%)	9 (41%)	7 (8%)	<0.001
Outcome				
Required hospitalization	2 (2%)	0 (0%)	2 (2%)	0.468
Death due to deterioration of infection	8 (7%)	4 (18%)	4 (5%)	0.032
30 days-mortality	14 (13%)	7 (32%)	7 (8%)	0.004

Predictors of hypoxemic respiratory failure due to home-care-acquired infection

Table 3 shows the results of the logistic regression analysis performed to determine the predictors of hypoxemic respiratory failure due to home-care-acquired infection. We included five potential confounding factors, which presented significant differences between the two cohorts in univariate analysis. As shown in Table 3, the significant predictors of respiratory failure were clinical diagnosis of lower respiratory infection, followed by initial treatment failure and malignant disease as the underlying disease.

**Table 3:** Predictors of hypoxemic respiratory failure due to home-care-acquired infection. OR: odds ratio; CI: confidence interval

	Hypoxemia (n=22 of 107)	Multivariate analysis	
		OR (95% CI)	p
Lower respiratory infection	18/55 (33%)	11.08 (2.66-46.15)	0.001
Initial treatment failure	6/12 (50%)	7.28 (1.31-40.41)	0.023
Malignant disease	12/17 (71%)	7.10 (1.96-25.64)	0.003
Dementia	6/44 (14%)	0.55 (0.13-2.39)	0.425
Age >80 years	6/54 (11%)	0.39 (0.10-1.56)	0.184

Comparison of clinical features in patients with hypoxemic respiratory failure due to home-care-acquired infection with those in patients with COVID-19 over 60 years old

For further investigation, we compared the clinical features between patients who developed hypoxemia due to home-care-acquired infection and those with COVID-19 over 60 years of age. As shown in Table 4, two parameters were significantly different: the presence of febrile co-habitants and the timing of hypoxemia development after the onset of infection.

**Table 4:** Clinical features of hypoxemia due to infection in home-care medication and COVID-19 in patients over 60 years old. COVID-19: coronavirus disease 2019; IPPV: invasive positive-pressure ventilation; NHF: nasal high flow

	Home-care-acquired infection (n=22)	COVID-19 >60 years (n=36)	p
Febrile co-habitant in simultaneous period	0 (0%)	31 (86%)	<0.001
Initiation of oxygen therapy from fever-onset			
Within 2 days	22 (100%)	2 (6%)	<0.001
3-6 days	0 (0%)	19 (52%)	<0.001
>7 days	0 (0%)	15 (42%)	<0.001
Demand for oxygen flow rate (L/min)			
Within 5 L/min	22 (100%)	18 (50%)	—
5-14 L/min	0 (0%)	5 (14%)	—
Require IPPV or NHF	0 (0%)	13 (36%)	—

The total COVID-19 cohort in this study included 60 patients (median age 72 (67-79), male/female, 32/28. The underlying diseases were as follows: dementia (15%), malignant tumor (2%), diabetes mellitus (25%), and hypertension (55%). Logistic regression analysis determined the predictors of hypoxemic respiratory failure in the COVID-19 cohort as follows: fever prolonged over 72 h after onset (odds ratio (OR), 10.29; p=0.007), hypertension (OR, 7.03, p=0.006), and male sex (OR, 4.21, p=0.037). Details of the clinical features of the patients in the COVID-19 cohort and the results of the logistic regression analysis are described in Tables 5, 6.

**Table 5:** Clinical features in patients with COVID-19 over 60 years old admitted to Toyama University Hospital. Continuous variables are reported as median (interquartile range (IQR) 25-75). Categorical variables are reported as numbers (percentages). COVID-19: coronavirus disease 2019

	Total (n=60)	Hypoxemia (n=36)	Non-hypoxemia (n=24)	P
Age, years	72 (67-79)	74 (68-78)	72 (66-79)	0.409
Sex (male/female)	32/28	25/11	7/17	0.002
Underlying disease				
Dementia	9 (15%)	4 (11%)	5 (21%)	0.302
Malignant tumor	1 (2%)	1 (3%)	0 (0%)	0.410
Diabetes mellitus	15 (25%)	11 (31%)	4 (17%)	0.224
Hypertension	33 (55%)	27 (75%)	6 (25%)	<0.001
Febrile co-habitant in simultaneous period	51 (85%)	31 (86%)	20 (83%)	0.768
Duration of fever (days)	7 (4-10)	8 (6-13)	5 (2-10)	0.001
Treatment				
Anti-viral therapy	31 (52%)	25 (69%)	6 (25%)	-
Steroid therapy	27 (45%)	26 (72%)	1 (4%)	-
Outcome				
Death due to deterioration of infection	6 (10%)	6 (17%)	0 (0%)	0.035
30 days-mortality	6 (10%)	6 (17%)	0 (0%)	0.035

**Table 6:** Predictors of hypoxemia due to COVID-19 in patients over 60 years old. COVID-19: coronavirus disease 2019

	Hypoxemia (n= 36 of 60)	Multivariate analysis	
		OR (95% CI)	P
Fever prolonged over 72h after onset	33/44 (75%)	10.29 (1.87-56.47)	0.007
Hypertension	27/33 (82%)	7.03 (1.77-27.94)	0.006
Male	25/32 (78%)	4.21 (1.09-16.18)	0.037

## Discussion

In the present study, we demonstrated that initial treatment failure and underlying malignant disease were strongly associated with the development of hypoxemia due to home-care-acquired infections other than COVID-19. Among the patients who developed hypoxemia, there was a significantly lower incidence of febrile co-habitant and earlier onset of hypoxemia, than in those with COVID-19 over 60 years of age who were admitted to a tertiary hospital.

According to the rapidly increasing global aging population, home-based primary care and hospice care are widely recognized as alternatives for in-hospital care [1]. By integrating palliative care into primary care, home-based medication can be beneficial for older adults or patients with end-stage illnesses by improving cost-effective healthcare with better medical utilization, reducing unnecessary care setting transitions, and supporting home-based death with a better quality end of life [13-15]. Among these medical settings, acute infection is one of the most frequent and critical issues, which occasionally results in hospital admission or death [7,8]. To date, there have been a few published findings related to home-care-acquired infections. In the USA, Dwyer et al. reported that 10-12% of individuals receiving home healthcare had an infection during the study period, and the most common types of infection were urinary tract infections, pneumonia, and cellulitis [16]. In Japan, Yokobayashi et al. reported that 67% of 229 fever events under home-care medication were improved at home with antibiotic administration, while 23% were improved with in-hospital medication. Mortality was increased to 10% (5% at home, 5% at hospital), and the leading causes of febrile events were lower respiratory infections (n=103, 45.0%) [7]. However, there is a lack of evidence on the underlying causes of hypoxemia due to home-care-acquired infections.

In our study, antibiotics were initiated as soon as possible after the onset of fever in order to avoid unnecessary admission, since emergent admission to the hospital was difficult due to insufficient medical resources during the early COVID-19 pandemic; therefore, 87% were prescribed fluoroquinolone and 31% were treated with intravenous antibiotics including ceftriaxone and tazobactam/piperacillin. Among them, 21% (22 patients) developed hypoxemic respiratory failure, which required emergent initiation of oxygen therapy. In our clinical setting, initiation of home oxygen therapy was available within several hours after hypoxemia development; therefore, hospital admission was unnecessary for both hypoxemia cases. Although 7.6% (eight patients) died after developing an infection, none had desired care-setting transition and could stay home as the final place of end-of-life care as determined by advanced care planning.

During this period, family visits were strictly prohibited in hospitals even with end-of-life care; thus, there was an increasing demand for home-based palliative care, and most patients and families selected home as the place of care at the end of life. In terms of providing quality palliative care, patient preferences for home-based death have recently increased; however, the decision for the final place of care is occasionally affected by several unwilling factors: higher functional status, inadequately controlled symptoms, and acute reversible events [17-19]. With home-based palliative care, the proportion of home deaths has been reported as 52-71% [3,17]. Based on our experience with a relatively high rate of home death, we suggest that hypoxemia due to home-care-acquired infection can be treated in home-care settings, with immediate application of antibiotics and home oxygen therapy. However, the results of the logistic regression analysis advocate caution for initial treatment failure as a risk factor for hypoxemia due to infection. Antibiotics and their frequency of dosage are substantially highly limited in most patients receiving home-based medication; ceftriaxone (CTRX) is the representative applicable drug that could be administrated intravenously, and tazobactam piperacillin intravenous (TAZ/PIPC IV) is only available in a few nursing houses with a nurse on duty at all time. Although there remains the risk of insulting anti-microbial resistant bacterial infection, immediate administration of antibiotics for febrile patients in a home-care setting might be the most effective choice in order to avoid unnecessary and unwilling care-setting transition, in particular when encountering a period with insufficient medical resources. Further investigation is considered necessary to establish appropriate antibiotic therapy in home-based medicine, from various viewpoints including infectious disease and palliative care.

During the COVID-19 pandemic, nursing homes have become epicenters for the transmission of SARS-CoV-2, with a high mortality rate of 10-50% [20,21]. Regarding the nature of the disease, the severity of COVID-19 increases with age; however, the analysis of 30 days mortality in hospitalized patients over 65 years old seemed to be similar to that observed in nursing homes [22,23]. In our regions, the outbreak in healthcare facilities sporadically occurred; however, it was difficult to conduct a research-aimed investigation for privacy reasons in those facilities, because several habitats died unexpectedly, possibly due to the COVID-19 outbreak brought by the care workers in the facilities. Moreover, there have been very few patients who developed COVID-19 in individual home settings. Therefore, we investigated the clinical features of COVID-19 patients who were admitted to a tertiary hospital in a comparison with those underlying home-care-acquired infections. Despite substantial limitations, the comparison indicated two distinct characteristics of hypoxemic infection under home-care settings: lower incidence of febrile co-habitant and earlier onset of hypoxemia compared with COVID-19 patients of similar age. In this study, most home-care-acquired infections were considered to be bacterial infections due to the sensitive recovery response after antibiotic administration. From several descriptive studies on the natural course of COVID-19 [24,25], respiratory failure typically occurs several days (at least three days) after the first symptoms, which is compatible with our COVID-19 cohort. Based on these findings, hypoxemia developed within 48 hours after fever onset might be a significant diagnostic aid for the immediate differentiation of non-COVID-19 infection in home-care settings.

Our study has several limitations. First, the small number of patients with a hypoxemic infection under home-based medication may have limited our ability to show differences in clinical features. Second, a large proportion of infections were clinically diagnosed without microbiological examination. Third, the examination for COVID-19 could not be performed in all cases, which might have resulted in underdiagnosed COVID-19 cases in the cohort of patients infected under home-based medication. Since microbiological examinations are mainly performed at external institutions in home-care settings, it is difficult to conduct clinical studies with confirmed diagnoses of each infection using bacterial culture or PCR for SARS-CoV-2. Fourth, there remained the slight possibility that co-infection with any bacteria and COVID-19 might be included as non-COVID-19 patients in our cohort. Fifth, the locations where the participants dwelled were different between the home-based medication cohort and the COVID-19 cohort, which affected the selection bias. Moreover, this study defined both those who dwelled in individual homes and nursing homes (homes for the elderly with/without health and welfare services) as the patients receiving home-based medication. In the Japanese health system, medication supplied in an elderly facility is integrated as ‘home-based medication’. These may have limited the results of our study, partly within the domestic context. However, we believe our work would be helpful for understanding the broad range of clinical practice for elderly or palliative care in home-care settings, which urgently demand epidemiological data on hypoxemic infections.

## Conclusions

In conclusion, this study demonstrated that hypoxemia due to home-care-acquired infection was strongly associated with initial treatment failure and underlying malignant disease, which were distinct features, possibly different from those underlying hypoxemia due to COVID-19 in the early pandemic era. Further investigation is necessary to establish appropriate management of hypoxemic failure under home-based medicine.
